# Palonosetron and aprepitant for the prevention of postoperative nausea and vomiting in patients indicated for laparoscopic gynaecologic surgery: a double-blind randomised trial

**DOI:** 10.1186/1471-2253-14-68

**Published:** 2014-08-10

**Authors:** Hyoung Yong Moon, Chong Wha Baek, Geun Joo Choi, Hwa Yong Shin, Hyun Kang, Yong Hun Jung, Young Cheol Woo, Jin Yun Kim, Seul Gi Park

**Affiliations:** 1 Department of Anesthesiology and Pain Medicine, College of Medicine, Chung-Ang University, Seoul, Republic of Korea

**Keywords:** Palonosetron, Aprepitant, Laparoscopic, Gynaecologic, PONV

## Abstract

**Background:**

Postoperative nausea and vomiting (PONV) is one of the most common postsurgical complications. Palonosetron, a 5-hydroxytryptamine receptor antagonist, is effective for PONV prevention. Herein, we compared palonosetron and aprepitant (a neurokinin-1 receptor antagonist) for PONV prevention in patients indicated for laparoscopic gynaecologic surgery.

**Methods:**

Ninety-three patients who were scheduled to undergo laparoscopic gynaecologic surgery under general anaesthesia were assigned to receive either a single intravenous injection of 0.075-mg palonosetron or 40-mg oral aprepitant in a double-blind randomised trial. The primary efficacy end points included complete response (visual analogue scale [VAS] nausea score <4 and no use of rescue therapy) 0–48 h after surgery. Nausea severity (0–10) and use of rescue therapy were monitored for 0–48 h. The secondary efficacy end points were the effect of aprepitant quantified using a 10-point VAS for pain, consumption of intravenous patient-controlled analgesia, and use of rescue analgesics.

**Results:**

Aprepitant was non-inferior to palonosetron in terms of complete response 0–48 hours after surgery (74% vs. 77%). At 0 and 2 h after administration, the nausea severity with 40-mg aprepitant was significantly lesser than that with 0.075-mg palonosetron (P < 0.05). At 6 and 24 h after administration, fentanyl consumption with 40-mg aprepitant was significantly lower than that with 0.075-mg palonosetron. Greater amounts of rescue analgesics were required in the aprepitant group.

**Conclusions:**

Palonosetron and aprepitant were both effective for PONV prevention in the patients indicated for laparoscopic gynaecologic surgery. The drugs can be used in combination for multimodal therapy because they bind to different receptors. More research is needed to evaluate the effects of aprepitant on pain management in humans.

## Background

Postoperative nausea and vomiting (PONV) is one of the most common postsurgical complications. It is caused by various factors such as the use of anaesthetics, the use of opioids for postoperative pain, the type of surgery, and patient characteristics [1]. PONV decreases patient satisfaction with surgical outcomes, extends the hospitalisation period because of delayed recovery, and causes fatal complications, including suture laceration, bleeding, increased intracranial pressure, aspiration pneumonia, dehydration, and electrolyte imbalance [2].

The vomiting centre in the brain stem, which is comprised of the reticular formation and nucleus tractus solitaries, can be activated directly via irritants or indirectly via 4 principal areas, namely the gastrointestinal tract, cerebral cortex and thalamus, vestibular region, and chemoreceptor trigger zone [3]. These regions contain high concentrations of opioid, dopamine, serotonin (or 5-hydroxytryptamine), histamine, and muscarini cholinergic receptor [4]. Various classes of medications, including serotonin receptor antagonists, dopamine receptor antagonists, and steroids, are currently used to prevent PONV.

Among such medications, palonosetron (Aloxi injection; CJ CheilJedang Corp., Seoul, Republic of Korea) is a long-acting, second-generation serotonin receptor antagonist that is one of the most commonly used antiemetics for PONV prevention. Substance P or the neurokinin-1 (NK_1_) receptor is also found in gastrointestinal vagal afferents and the nucleus tractus solitaries [5]. Its antagonist, aprepitant (Emend capsule; MSD Korea Ltd., Seoul, Republic of Korea), was developed to prevent chemotherapy-induced nausea and vomiting, similar to other antiemetic drugs. Its efficacy in PONV prevention was recently demonstrated [6,7].

The primary aim of this study was to confirm the equivalent effects of palonosetron and aprepitant on PONV prevention. Eriksson and Korttila [8] reported that 80% of patients who underwent gynaecologic laparoscopy developed PONV. Apfel et al. [9] suggested the following PONV risk factors in adult patients who underwent general anaesthesia: female sex, non-smoking status, history of PONV and motion sickness, and perioperative opioid use. This study compared the effects of 2 widely used drugs for PONV prevention, namely palonosetron and aprepitant, in female patients who were indicated to undergo gynaecologic surgery.

Substance P is a neurotransmitter that is secreted when a strong stimulus occurs in the body, and its function in pain-related signalling is well documented. An animal study demonstrated the efficacy of aprepitant in controlling pain [10]. Based on the study results, aprepitant was suggested to affect postoperative pain and the amount of opioids needed for pain control. Therefore, the present study additionally investigated the effect of aprepitant on pain.

## Methods

This study was conducted with patients, American Society of Anesthesiologists physical status rating 1–2 and aged 20–60, who were scheduled to undergo laparoscopic gynaecologic surgery. Exclusion criteria were patients who were pregnant; weighed <45 or ≥100 kg; were smokers; and had a history of PONV, other serious medical ailment of the cardiovascular system, kidney, or liver, or a hepatic disorder. This study was approved by the Chung-Ang University Hospital Institutional Review Board and registered at the Australia-New Zealand Clinical Trials Registry (ACTRN12613000902796). The study objective, methods, and period, and the expected adverse events were explained to the patients before obtaining their consent for participation. Before surgery, the patients were also educated on the visual analogue scale (VAS), a tool by which nausea and pain are rated on a scale of 0 to 10, and the intravenous patient-controlled analgesia (IV-PCA), which would be used postoperatively.

The patients were divided into 2 groups using a random number generator in Microsoft Excel. The aprepitant group (group A) was given 40 mg of aprepitant with 30 mL of water orally, 90 min before anaesthesia induction. The patients were informed that the aprepitant was a premedication for their operation and were unaware that it was a study variable. All the patients in both groups received 0.2 mg of glycopyrrolate intramuscularly, and the standard monitoring methods, which included electrocardiography, non-invasive blood pressure assessment, and pulse oximetry, were initiated after the patients entered the operating room. First, 60-μg/kg midazolam and 2-mg/kg propofol were administered intravenously, followed by 0.6-mg/kg rocuronium after confirmation of the patients’ loss of consciousness. Thereafter, intubation was performed. In the palonosetron group (group P), patients blinded to their group status received 0.075 mg of palonosetron intravenously immediately after endotracheal intubation, whereas the patients in group A received an equal volume of normal saline. An independent anaesthesia assistant who was not involved in either intraoperative or postoperative management prepared the study medications. Desflurane with oxygen/nitrous oxide in 0.5 FiO_2_ was administered at a 1.5–2 minimum alveolar concentration to maintain anaesthesia, and the ETCO_2_ was maintained at 35–40 mmHg. For postoperative pain management, nefopam (20 mg) was diluted in 100 mL of normal saline and administered intravenously for 30 min, 10 min before surgery. Using automated IV-PCA (Automed 3300; Ace Medical, Seoul, Republic of Korea), 20-μg/kg fentanyl was diluted in normal saline for a total volume of 100 mL, set at a 0.2-μg/kg bolus and a 15-min lockout interval. At the end of surgery, 0.004-mg/kg glycopyrrolate and 0.2-mg/kg pyridostigmine were administered intravenously to reverse any residual neuromuscular block after restoration of spontaneous breathing in all patients. All the anaesthetic procedures were performed by a single anaesthesiologist who was blinded to the patient group allocation.

PONV treatment completion was defined as a VAS nausea score <4 for 48 h after surgery or non-use of additional antiemetic drugs during this period. The VAS score was used to quantify the severity of nausea in the recovery room and 2, 6, 24, and 48 h after surgery. When a patient experienced nausea at a VAS score >4 with retching or vomiting, 10 mg of metoclopramide was administered intravenously. When symptoms did not improve at follow-up, 5 mg of dexamethasone was administered intravenously.

Postoperative pain management was standardised, and patients were trained to press the IV-PCA button when they experienced pain. The VAS score was used to represent the pain that patients experienced in the recovery room and 2, 6, 24, and 48 h after surgery, and their consumption of fentanyl in the IV-PCV was checked. If the VAS score was ≥4, an additional 50 μg of fentanyl was administered and recorded. Postoperative management and data collection were conducted according to the study protocol by another anaesthesiologist and a trained member of the research group, respectively, who were blinded to the patient groupings. The patients remained unaware of their group affiliation until study completion.

This study aimed to demonstrate that aprepitant is non-inferior to palonosetron in preventing PONV. A pilot study was conducted among 40 patients who received palonosetron, which reported a 35% incidence of PONV 48 h after surgery. When the non-superiority margin of aprepitant was set at 25% with an α value of 0.05 and statistical power of 80%, the required number of patients for each group was 46. Considering that <10% of the patients were lost to follow-up, a total of 100 patients were enrolled in the present study.

Repeated-measures analysis of variance was used for the statistical analysis. For continuous variables, a *t* test was used; for discrete variables, chi-square or Fisher exact tests were used. PASW Statistics version 18.0 (SPSS Inc., IBM Corporation, Chicago, IL, USA) was used for the analysis, and P < 0.05 was considered statistically significant.

## Results

**Figure 1:**
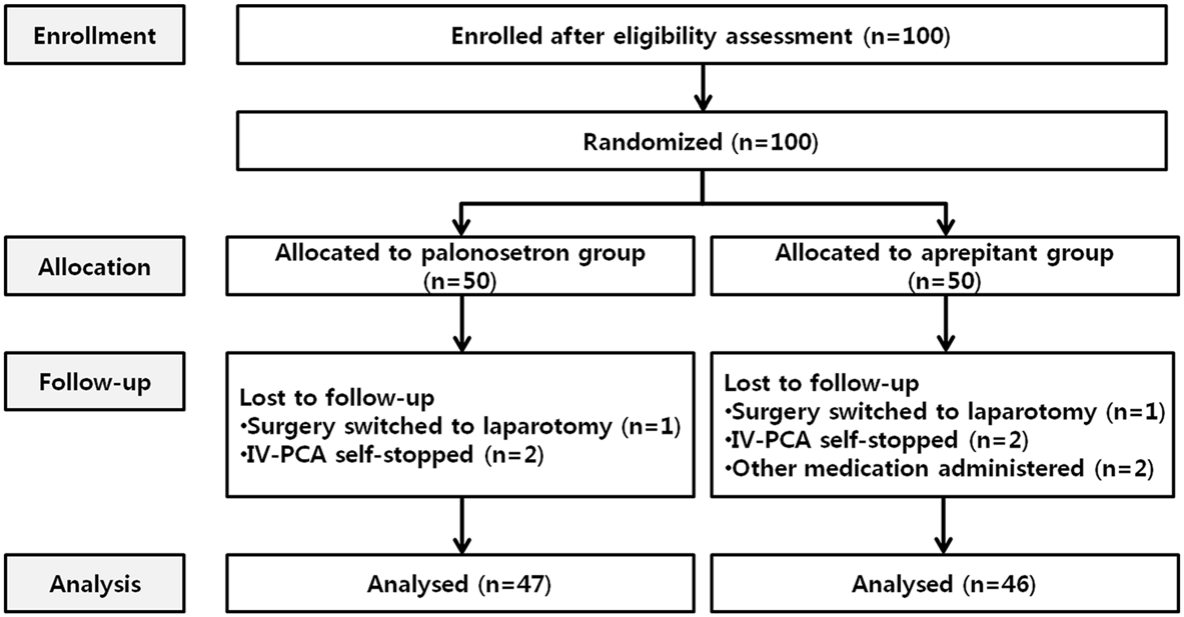
CONSORT diagram.

This study was conducted between October 2011 and September 2013 at the Chung-Ang University Hospital, Seoul, Republic of Korea. Among the 100 subjects enrolled in this study, 93 were included in the analysis, because 3 patients voluntarily stopped using IV-PCA, 2 switched to laparotomy during the laparoscopy, and 2 received other drugs in the ward (Figure 1). No significant differences were observed in the patients’ demographic data between groups P and A, including the surgical duration, age, height, and weight (Table 1).

**Table 1:** Characteristics of the patients who underwent laparoscopic gynaecologic surgery and antiemetic therapy with either 0.075 mg of palonosetron (group P) or 40 mg of aprepitant (group A)

	**Group P (n = 47)**	**Group A (n = 46)**
Operative time, minutes (mean ± SD)	79.2 ± 42.2	71.5 ± 37.7
Age, years (mean ± SD)	37.6 ± 8.0	37.9 ± 11.1
Height, cm (mean ± SD)	159.6 ± 5.1	160.5 ± 5.4
Weight, kg (mean ± SD)	54.8 ± 5.8	56.2 ± 5.6

The data are expressed as mean ± SD.

No significant differences were observed between groups P and A.

**Figure 2:**
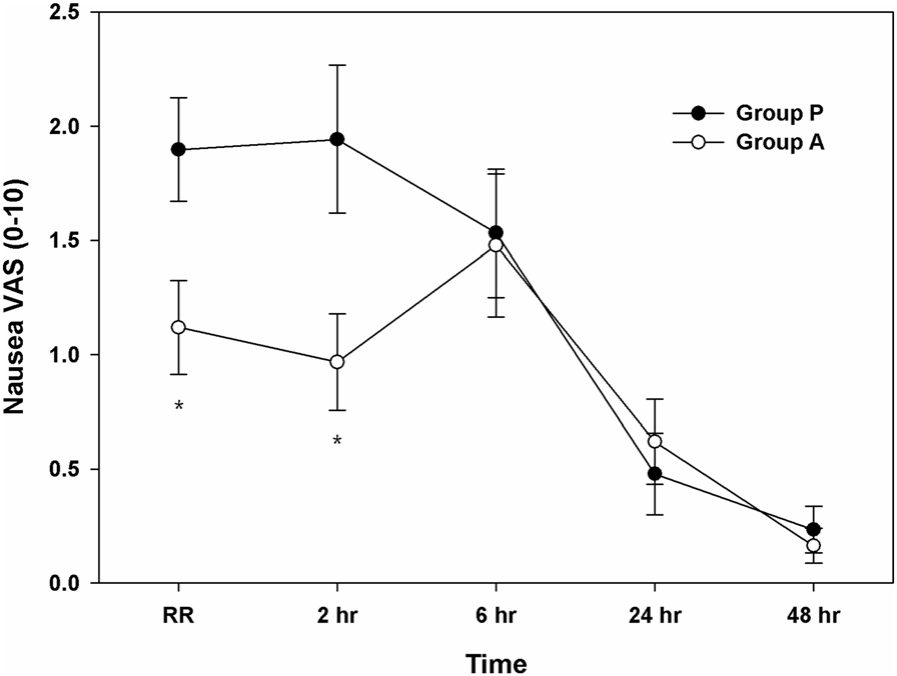
**Severity of nausea over 48 postoperative hours, graded using a 10-point visual analogue scale (VAS), in the female patients who underwent laparoscopic gynaecologic surgery.** The data are expressed as mean ± standard error values of the mean. *P < 0.05, compared with group P. Group P was given 0.075 mg of palonosetron intravenously, whereas group A was given 40 mg of oral aprepitant.

Thirteen of the 47 patients (27.7%) in group P and 13 of the 46 patients (28.2%) in group A received metoclopramide as an antiemetic medication, of which 2 patients in group P and 1 patient in group A received dexamethasone. The treatment completion rates were 72.3% and 71.8%, respectively, without significant difference (Table 2). The nausea intensity in the recovery room and 2 h after surgery assessed using the 10-point VAS was significantly lower in group A (11.2 ± 2.1 and 9.7 ± 2.1, respectively) than in group P (19.0 ± 2.2 and 19.4 ± 3.5, respectively; P < 0.05). However, the results at 6, 24, and 48 h after surgery did not differ significantly (Figure 2).

**Table 2:** Rescue antiemetic administration

**Postoperative time (h)**	**Group P (n = 47)**	**Group A (n = 46)**
0−2	10	5
2−6	4	7
6−24	1	2
24−48	0	0
Total incidence	15	14
Total patients	13	13

The data represent the number of patients. Group P was given 0.075 mg of palonosetron intravenously, whereas group A was given 40 mg of oral aprepitant. The total number of patients excludes the number of patients with repeated administration (2 patients in the palonosetron group and 1 patient in the aprepitant group). No significant differences were observed between groups P and A.

**Figure 3:**
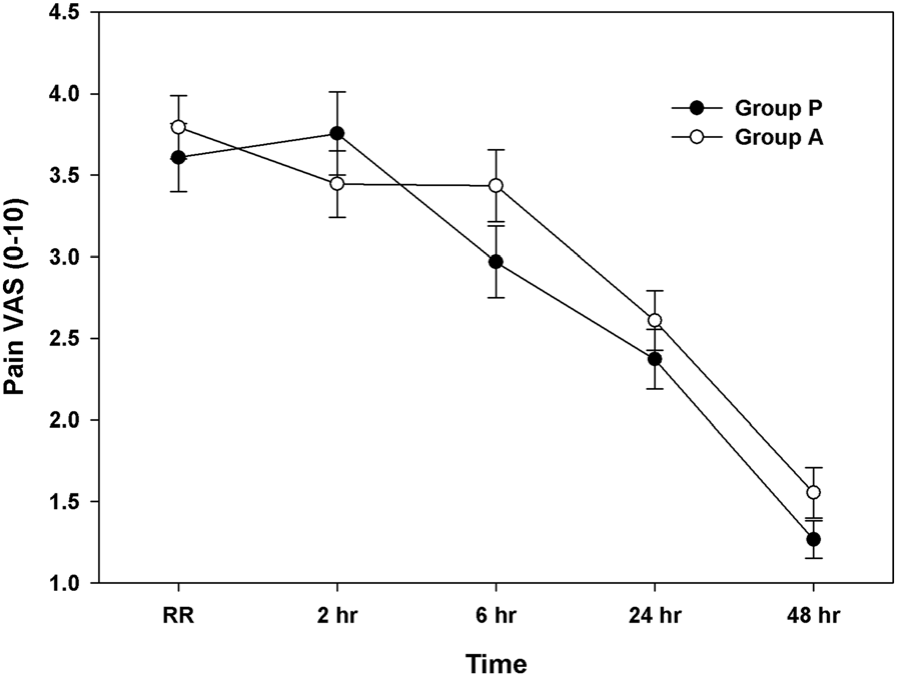
**Severity of pain over 48 postoperative hours in the female patients who underwent laparoscopic gynaecologic surgery, graded using a 10-point visual analogue scale (VAS).** The data are expressed as mean ± standard error values of the mean. Group P was given 0.075 mg of palonosetron intravenously, whereas group A was given 40 mg of oral aprepitant.

**Figure 4:**
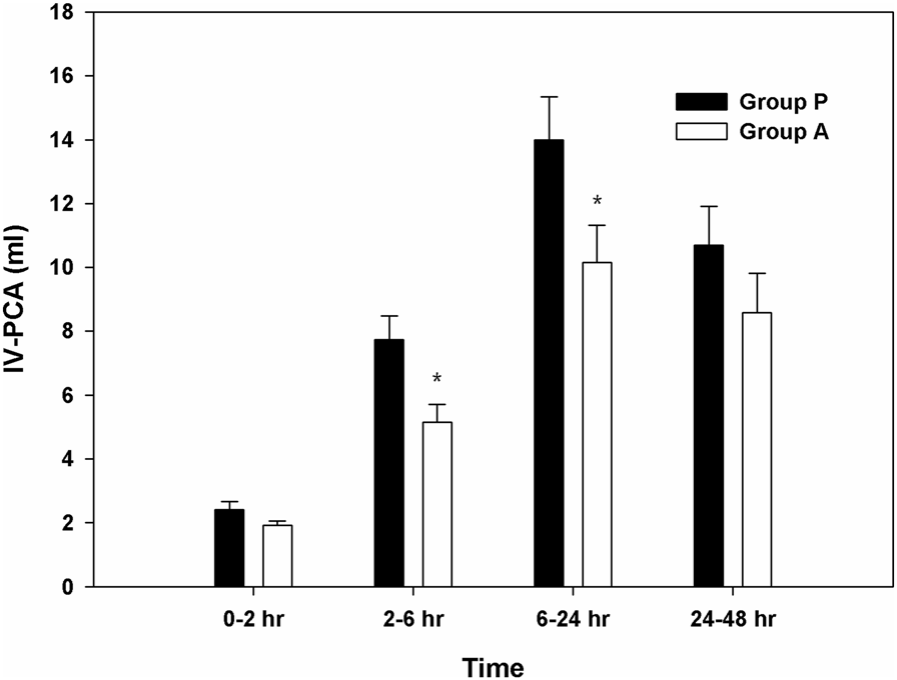
**Fentanyl consumption over 48 postoperative hours in the female patients who underwent laparoscopic gynaecologic surgery.** The graph shows the changes in fentanyl consumption according to the type of drug administered. The data are expressed as mean ± standard error values of the mean. *P < 0.05, compared with the palonosetron group. Group P was given 0.075 mg of palonosetron intravenously, whereas group A was given 40 mg of oral aprepitant.

The pain intensity, also measured using a 10-point VAS, was also not significantly different throughout the study period (Figure 3). Fentanyl consumption via automated IV-PCA was significantly lower in group A than in group P at 2 and 6 h after surgery (Figure 4). Finally, 17 of the 47 patients in group P (36.2%) and 20 of the 46 patients in group A (43.5%) received additional fentanyl. One patient in each group received fentanyl twice, and 4 patients in group P and 3 in group A received fentanyl thrice. No significant differences were observed in the incidence and number of additional fentanyl administrations between the 2 groups (Table 3).

**Table 3:** Rescue analgesic administration

**Postoperative time (h)**	**Group P (n = 47)**	**Group A (n = 46)**
0−2	15	13
2−6	7	9
6−24	4	4
24−48	0	1
Total incidence	26	27
Total patients	17	20

The data represent the number of patients. Group P was given 0.075 mg of palonosetron intravenously, whereas group A was given 40 mg of oral aprepitant. The total number of patients excludes the number of patients with repeated administration (5 patients in the palonosetron group and 4 patients in the aprepitant group). No significant differences were observed between groups P and A.

## Discussion

The PONV treatment completion rate was 72.3% (34/47) in group P and 71.8% (33/46) in group A. These data show that aprepitant has the same PONV prevention effect as palonosetron. Previous studies showed a wide range of prevention effects of antiemetic drugs (22.9 − 77.8%), depending on which drugs were administered for PONV prevention in patients indicated for laparoscopic surgery [11]. Park et al. [12] reported a 66% treatment completion rate when palonosetron was administered for PONV prevention in patients indicated for gynaecologic laparoscopic surgery, and Jung et al. [13] reported 56% and 63% treatment completion rates in the 80- and 125-mg aprepitant groups, respectively.

The subjects in the present study were female non-smokers who received opioids for postoperative pain and were included in the high-PONV-risk group. Despite the risk factors, this study showed a higher PONV treatment completion rate than other studies. One explanation for this difference is that midazolam and propofol, which were used to induce anaesthesia, might have helped prevent PONV. Even a small dose of propofol is known to have an antiemetic effect, and a previous study reported that administration of 10 mg of propofol successfully treated PONV [14]. Propofol is associated with a lower PONV incidence than inhalation anaesthetics [15,16], and Kim et al. [17] reported that combined administration of a serotonin receptor antagonist and midazolam was effective in PONV prevention. As such, both drugs were used in all the subjects in this study, which may explain the low incidence of PONV compared with that in other studies. Fentanyl is an opioid and represents the major cause of PONV. Unlike in the studies by Park et al. [12] and Jung et al. [13], in this study, the basal infusion dose of fentanyl was not set via IV-PCA. Therefore, its dose was reduced when our patients experienced less pain, which might also explain the high PONV treatment completion rate in this study.

For the patients who still experienced retching or vomiting and had a VAS score of ≥4 even after administration of drugs for PONV prevention, multiple drug administration was recommended for prevention [18]. Lee et al. [19] recently reported more significantly reduced nausea and vomiting in patients treated with a combination of ramosetron, a serotonin receptor antagonist, and aprepitant than in patients treated with aprepitant alone for 24 h. Aprepitant does not exhibit affinity for 5-hydroxytryptamine, dopamine, or steroid receptors, to which retching-related neurotransmitters bind [6]. As such, palonosetron and aprepitant can be administered together effectively to patients at high risk of PONV owing to their different action sites. Palonosetron is administered intravenously, whereas aprepitant is administered orally. Patients who are subjected to general anaesthesia have to undergo a period of fasting after surgery, which renders the postoperative administration of aprepitant difficult. Thus, preoperative oral administration of aprepitant and perioperative intravenous administration of palonosetron in patients with nausea and vomiting would likely result in a synergistic effect if the 2 drugs show similar effects when administered separately.

In this study, a lower dose of aprepitant (40 mg) was used than in the study of Jung et al. [13]. A previous study that provided the basis for determining the aprepitant dose in this study reported that 80 mg of aprepitant was suitable for patients undergoing chemotherapy, whereas 40 mg was sufficient for PONV prevention [6]. Furthermore, Diemunsch et al. [20] reported that 40 mg of orally administered aprepitant was more effective in PONV prevention than ondansetron. In this study, we also observed that 40 mg of aprepitant administered preoperatively was effective for PONV prevention.

Aprepitant selectively inhibits substance P from binding to the NK_1_ receptor [21]. Substance P expression is observed more often in the dorsal root than in the ventral root of the spinal cord. Lembeck suggested that substance P is a primary sensory neurotransmitter [22]. Substance P is also observed in smaller and unmyelinated sensory fibres [23]. It is involved in pain stimulus formation and is known to transmit pain. An animal study reported decreased pain when aprepitant was administered [10]. However, Hill et al. [24] reported that the effect of NK_1_ receptor blockers on pain control remains to be fully established in humans. In this study, the VAS was used to measure postoperative pain intensity, although the results in the palonosetron and aprepitant groups did not significantly differ. The fentanyl consumption in IV-PCA was significantly lower in group A than in group P 6 and at 24 h after surgery. This result was similar to that of the study of Katuta et al. [25], in which the analgesic requirement was lower even if the pain severity did not significantly differ. A greater number of patients in group A (43.5%, 20/46) received an additional administration of analgesic than that in group P (36.2%, 17/47), although the difference was not statistically significant. The effect of aprepitant on pain reduction was difficult to demonstrate.

The present study had several limitations. This study used propofol and midazolam to induce anaesthesia, which might have affected the PONV incidence, as described previously. However, although their pure antiemetic effects were not measured, the same doses of propofol and midazolam were used in the 2 groups to enable comparison of the effectiveness of palonosetron and aprepitant in PONV prevention. The absorption and distribution of aprepitant can differ depending on the dose and treatment duration. It takes approximately 3 h for 40 mg of orally administered aprepitant to reach its maximum blood concentration [6]. In this study, however, aprepitant was administered 90 min before anaesthesia induction, and the surgery duration differed among patients. As such, the maximum blood concentration of aprepitant might not have been reached in some of the patients at surgery completion. However, the VAS nausea scores were lower in group A until 2 h after surgery, which shows that the timing of aprepitant administration did not significantly affect the results. Moreover, complete allocation concealment and double-blinding were difficult because of the obviously different timing and drug administration routes between the groups. To ensure that this did not induce any bias in obtaining study outcomes, we adhered to a rigorous study protocol, as described in the Subjects and Methods. Finally, in the planning stage of this study, the appropriate number of subjects was determined based on the PONV treatment rate. More subjects could have been enrolled for more accurate pain study results. The effect of aprepitant on pain control can be assessed accurately in future studies if the appropriate number of subjects is determined based on pain intensity.

## Conclusions

In this study, aprepitant was as effective as palonosetron for PONV prevention in patients who underwent gynaecologic laparoscopic surgery. However, the effectiveness of aprepitant for postoperative pain control in relation to substance P warrants further investigation.

## Competing interests

The authors declare that they have no competing interests.

## Authors’ contributions

HYM was the primary individual who wrote the manuscript and performed the data analysis as the first author. CWB was the primary investigator; designed and conducted the study, performed the data analysis, and prepared the manuscript. GJC assisted in the preparation and translation of the manuscript. HYS was responsible for grouping the patients in the study and the preparation of the study medication. The author also assisted in the manuscript preparation. HK helped design and analyse the statistical study and assisted in the manuscript preparation. YHJ administered general anaesthesia to the study patients. YCW was the clinical advisor for the research study and manuscript preparation. JYK was the clinical advisor for the decision support rules, research study, and manuscript preparation. SGP explained the study design to the patients and collected the data. All authors read and approved the final manuscript.

## Pre-publication history

The pre-publication history for this paper can be accessed here:

http://www.biomedcentral.com/1471-2253/14/68/prepub
